# Supplementary material for: Ultrasound and Microbubbles Mediated Bleomycin Delivery in Feline Oral Squamous Cell Carcinoma—An In Vivo Veterinary Study
Source: Pharmaceutics. 2023 Apr 6;15(4):1166. doi: 10.3390/pharmaceutics15041166 (PMC10142092; doi:10.3390/pharmaceutics15041166)
Supplement: Supplementary file 1 [file pharmaceutics-15-01166-s001.zip › pharmaceutics-2201791-supplementary.pdf]

Ultrasound and Microbubbles Mediated Bleomycin Delivery in Feline Oral Squamous Cell Carcinoma—An In Vivo Veterinary Study

Josanne S. de Maar, Maurice M. J. M. Zandvliet, Stefanie Veraa, Mauricio Tobón Restrepo,

Chrit T. W. Moonen and Roel Deckers

Supplementary information:

Supplementary figure S1 Peak intensity (1/3)

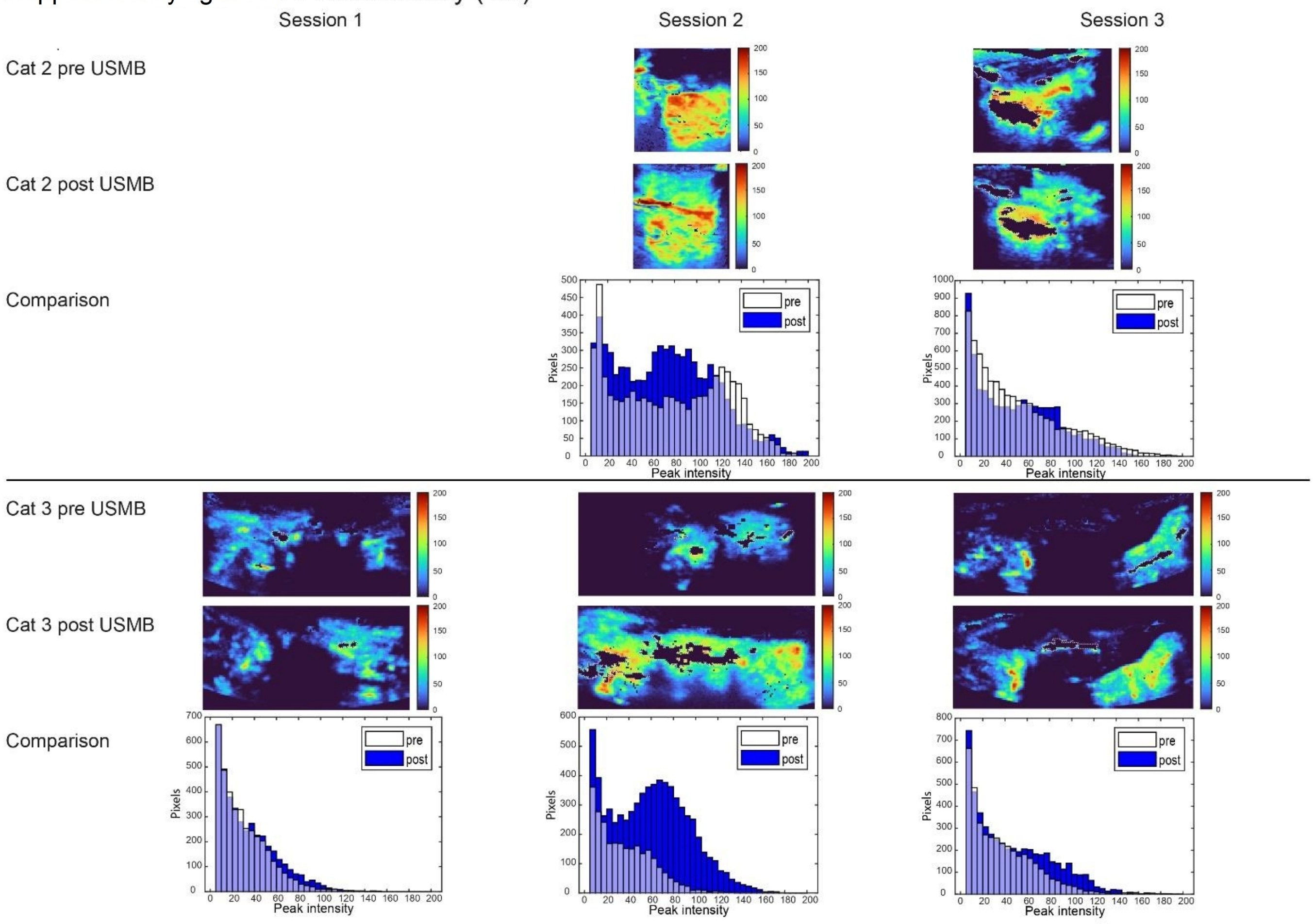

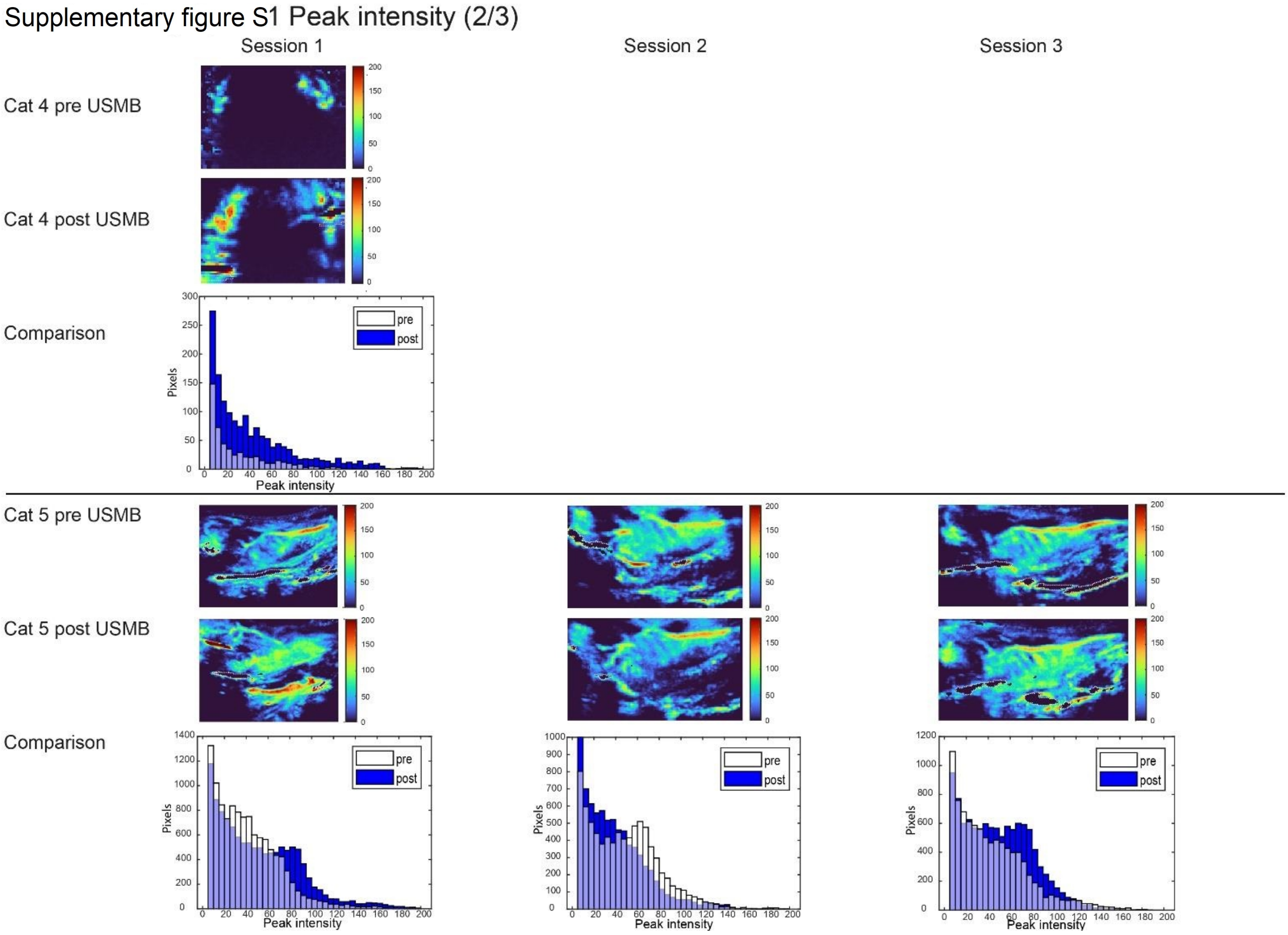

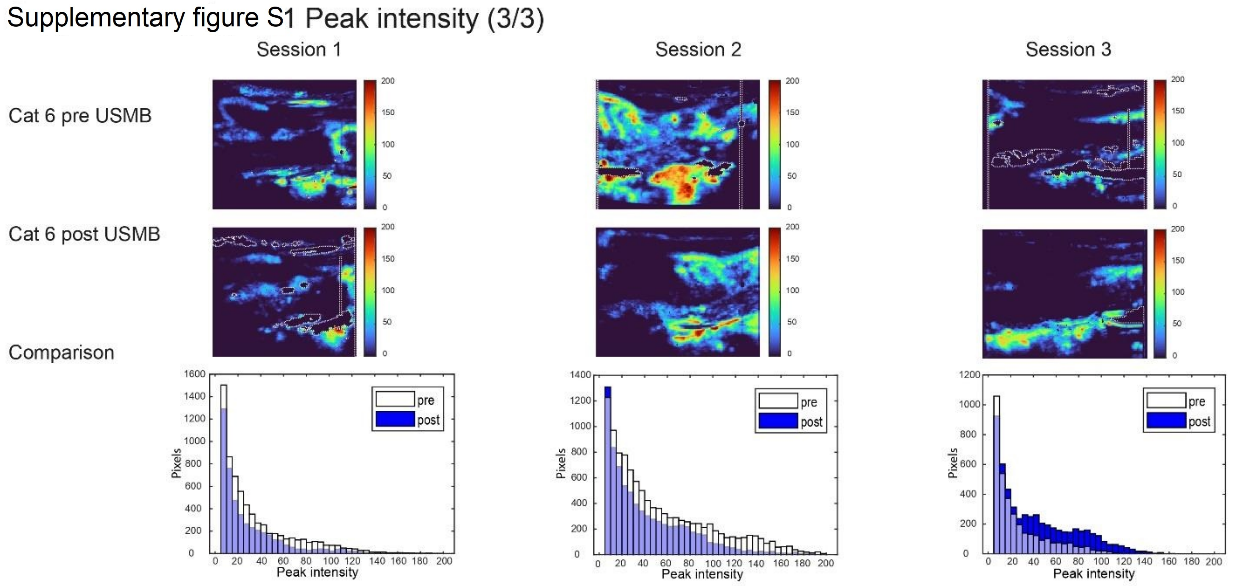

Figure S1. Contrast enhanced ultrasound (CEUS) parametric maps of cats 2-6, visualizing peak intensity (PI), before (top) and after (bottom) USMB therapy. For each cat the three treatment sessions are depicted from left to right. CEUS was available for twelve treatment sessions. Pixels with high signal intensity before administration of microbubbles (e.g. regions containing bone) were set to zero and delineated with white dotted lines. Below the two maps PI is compared between before (white) and after (blue) USMB in a histogram, excluding pixels with a PI < 5. Note that the range of the y-axes differs between treatment sessions. ROIs pre and post USMB are identical in size within one treatment session and matched in position as much as possible. CEUS parameters were kept

constant between treatment sessions, except in treatment session 2 of cat 3 when gain inadvertently changed from 45% (before USMB) to 49% (after USMB). Based on visual interpretation of the data PI increased in six out of nine treatment sessions.

Supplementary figure S2 Time to peak (1/3)

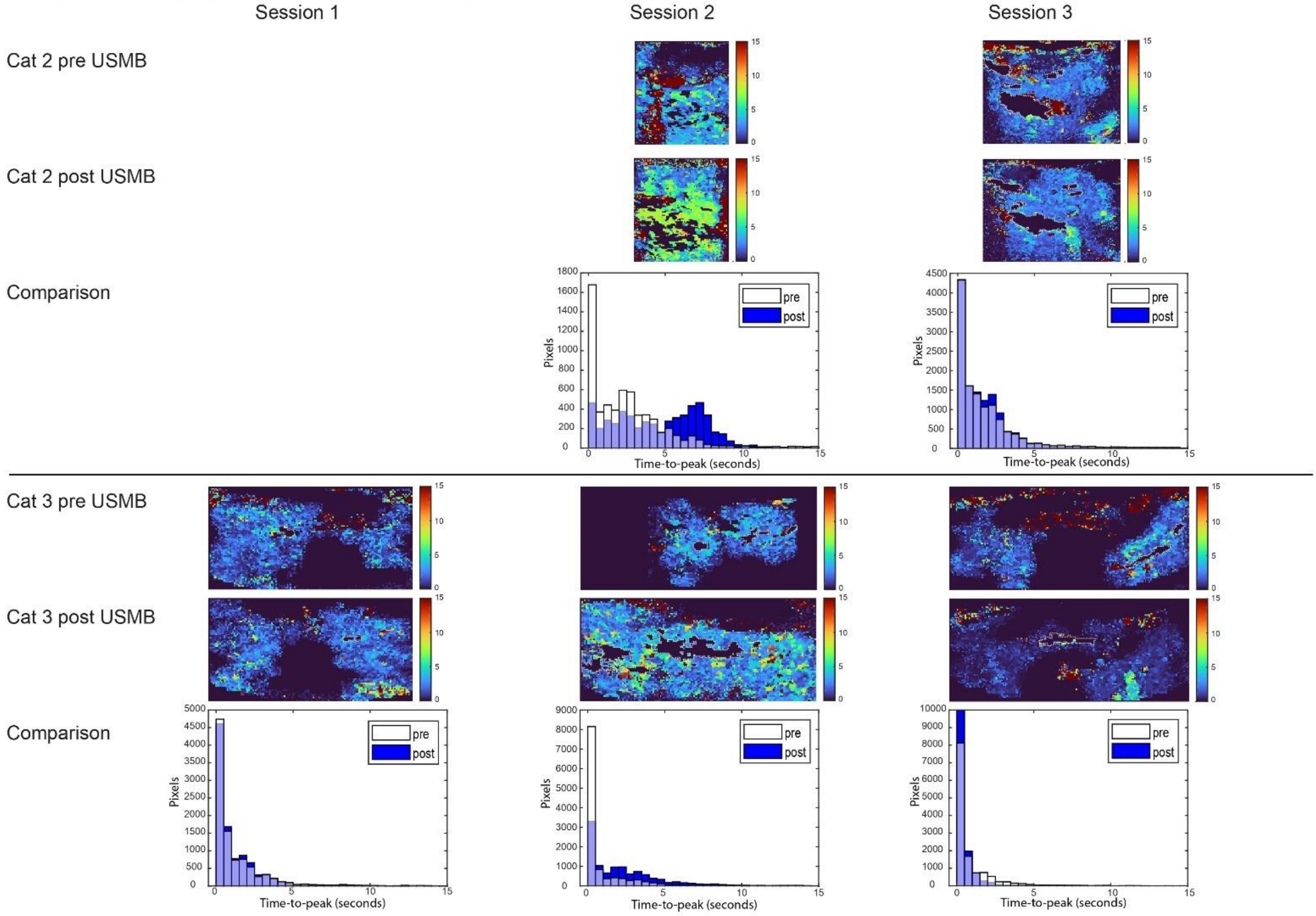

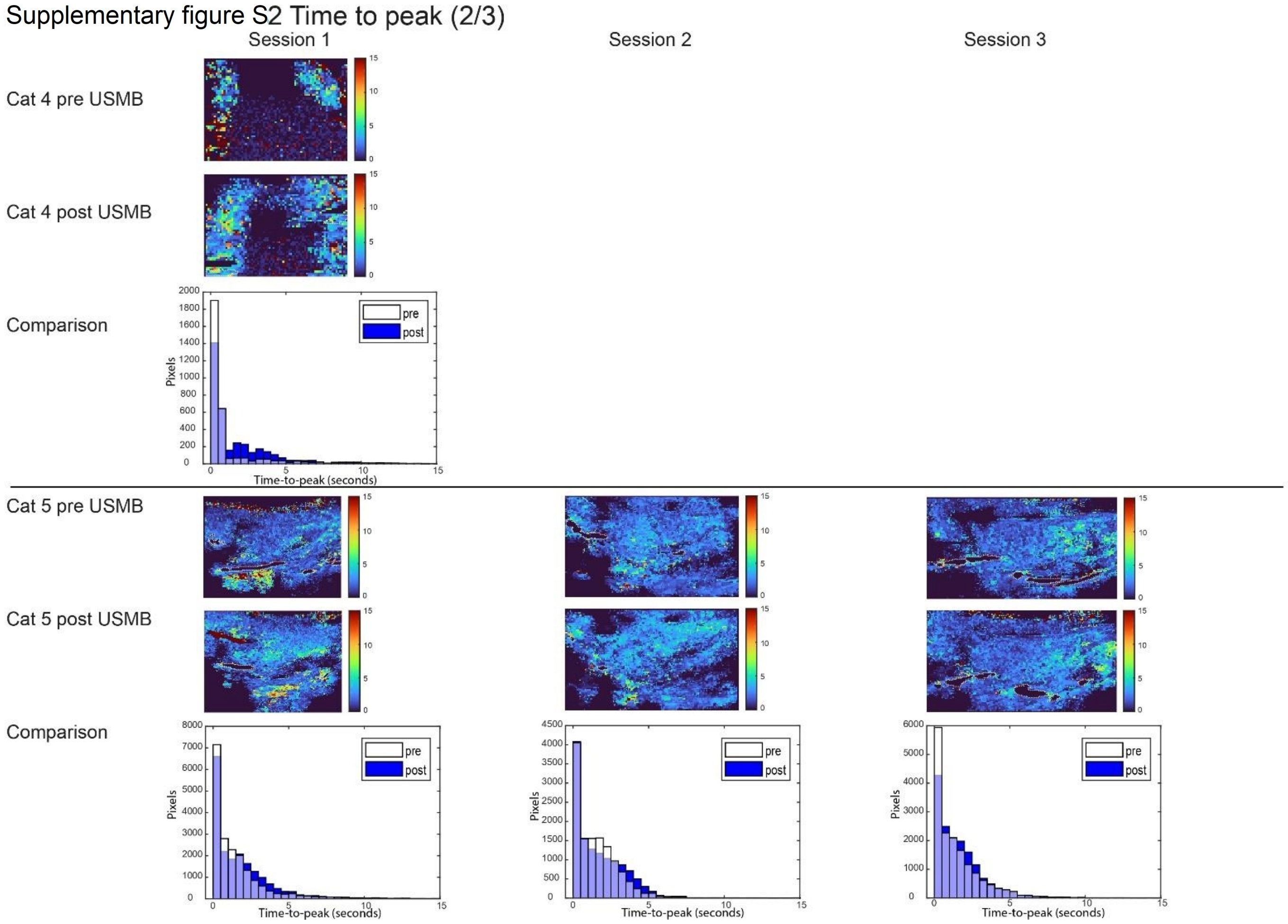

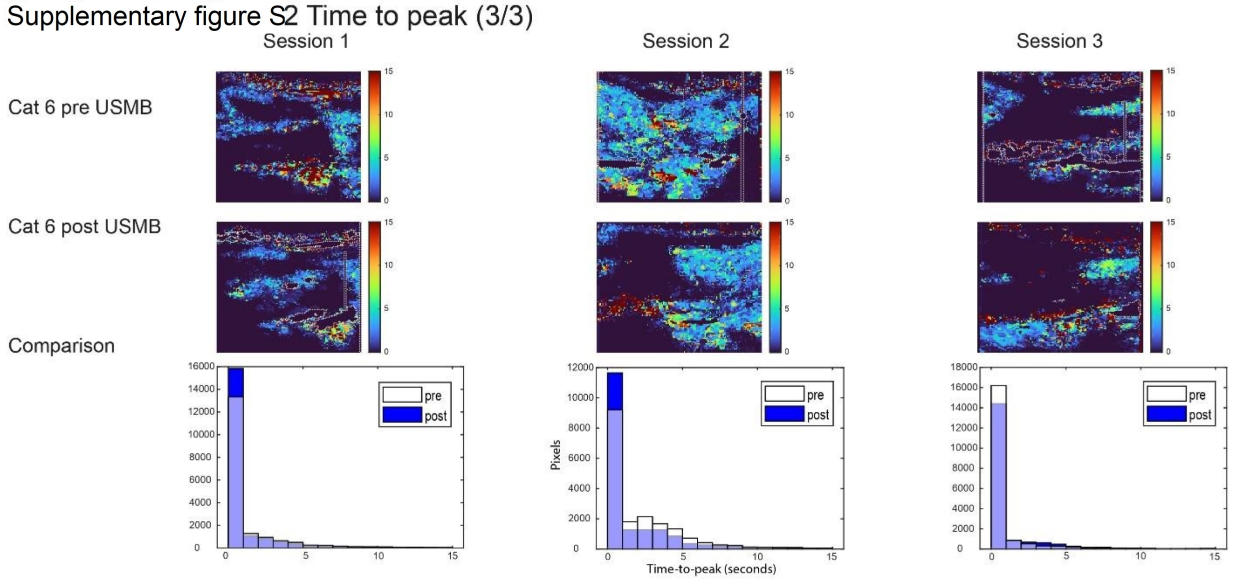

Figure S2. Contrast enhanced ultrasound (CEUS) parametric maps of cats 2-6, visualizing time-to-peak (TTP), before (top) and after (bottom) USMB therapy. For each cat the three treatment sessions are depicted from left to right. CEUS was available for twelve treatment sessions. Pixels with high signal intensity before administration of microbubbles (e.g. regions containing bone) were set to zero and delineated with white dotted lines. Below the two maps TTP is compared between before (white) and after (blue) USMB in a histogram. Note that the range of the y-axes differs between treatment sessions. ROIs pre and post USMB are identical in size within one treatment session and matched in position as much as possible. CEUS parameters were kept constant between treatment sessions,

except in treatment session 2 of cat 3 when gain inadvertently changed from 45% (before USMB) to 49% (after USMB). Based on visual interpretation of the data we did not observe a trend in changes of TTP.

Supplementary figure S3 Area under the curve (1/3)

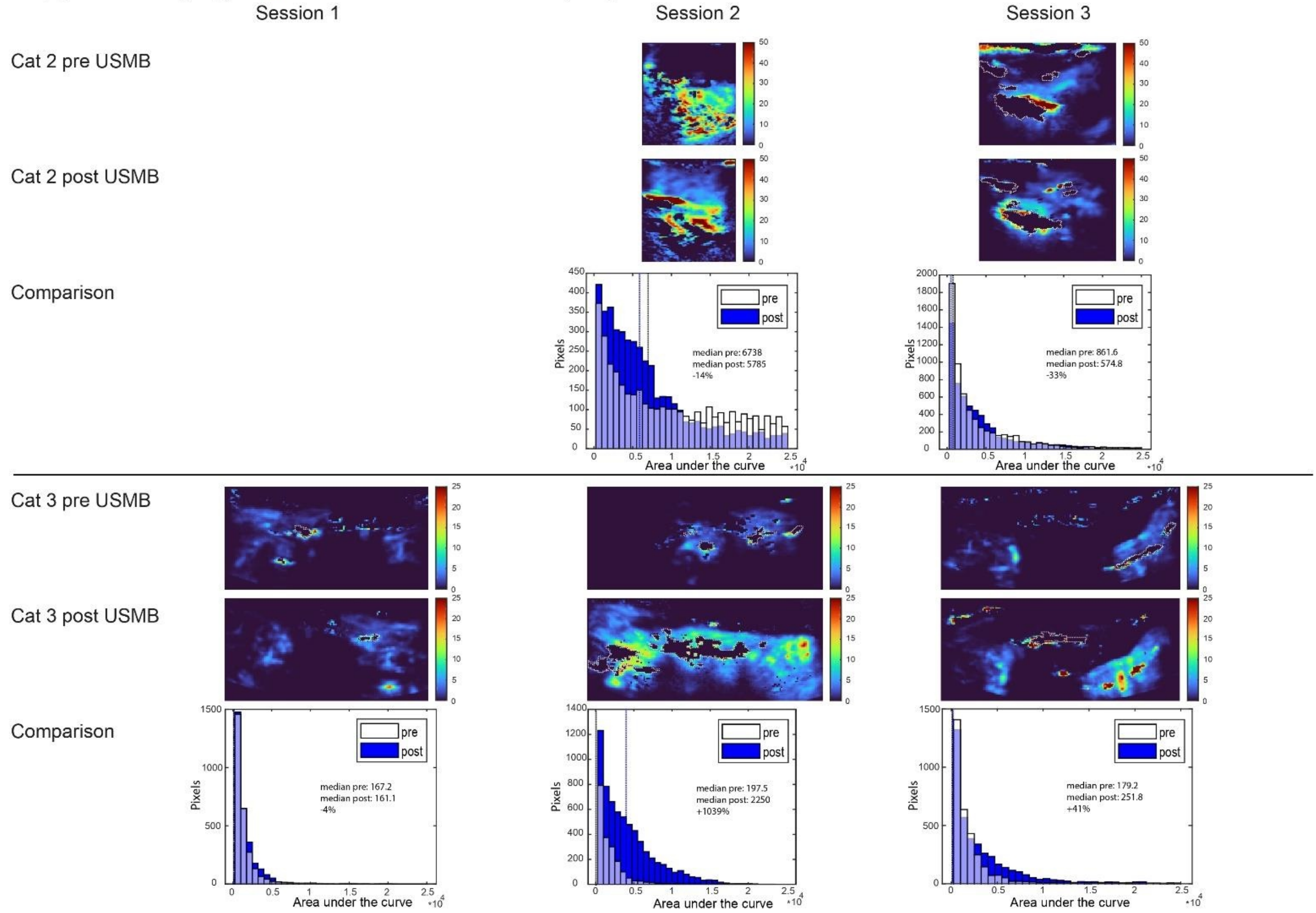

Supplementary figure S3 Area under the curve (2/3)

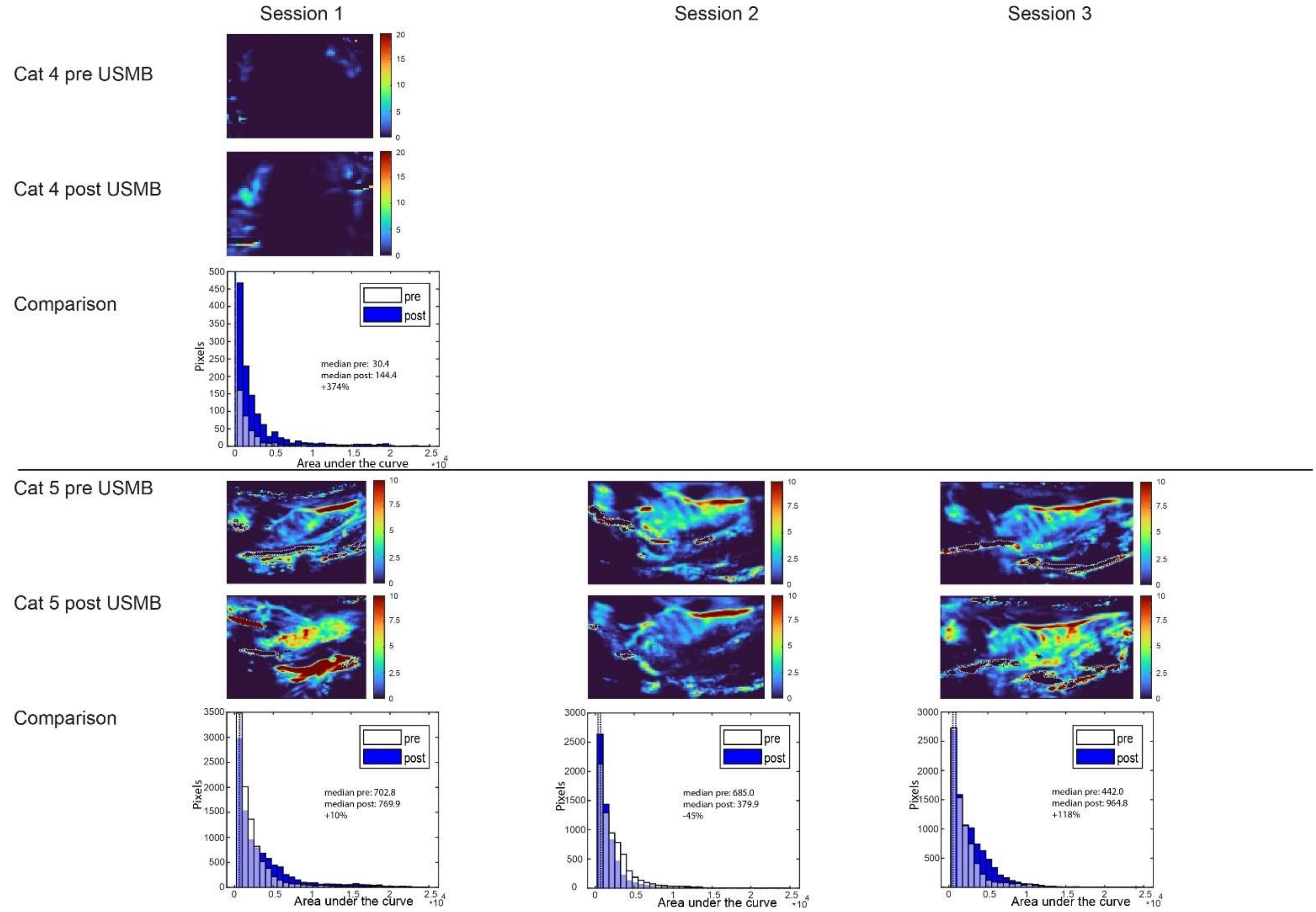

Supplementary figure S3 Area under the curve (3/3)

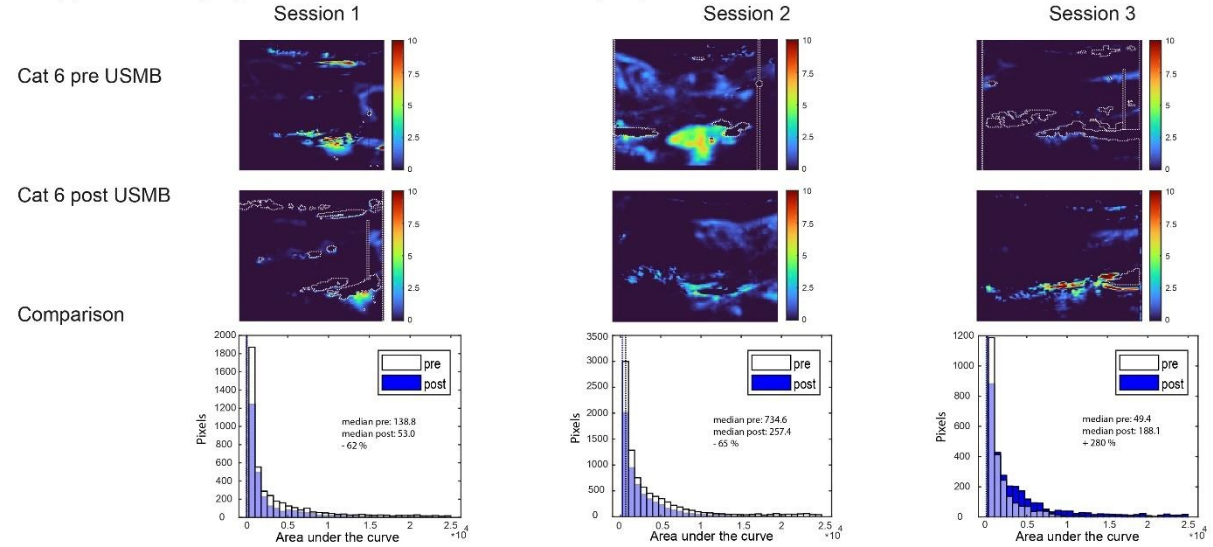

Figure S3. Contrast enhanced ultrasound (CEUS) parametric maps of cats 2-6, visualizing area under the curve (AUC), before (top) and after (bottom) USMB therapy. For each cat the three treatment sessions are depicted from left to right. CEUS was available for twelve treatment sessions. Pixels with high signal intensity before administration of microbubbles (e.g. regions containing bone) were set to zero and delineated with white dotted lines. Below the two maps AUC is compared between before (white) and after (blue) USMB in a histogram, excluding pixels with an AUC < 250. Median AUC values before and after USMB and the relative difference are described in the histogram. Note that colour scales and the range of the y-axes differ between treatment sessions. ROIs

pre and post USMB are identical in size within one treatment session and matched in position as much as possible. CEUS parameters were kept constant between treatment sessions, except in treatment session 2 of cat 3 when gain inadvertently changed from 45% (before USMB) to 49% (after USMB). The median AUC in the ROI decreased in six out of twelve treatment sessions (median decrease 39%, range -4 to -65%) while it increased in six out of twelve treatment sessions (median increase 199%, range 10 to 1039%).
